# On the Role of Mentalizing Processes in Aesthetic Appreciation: An ERP Study

**DOI:** 10.3389/fnhum.2015.00600

**Published:** 2015-11-13

**Authors:** Susan Beudt, Thomas Jacobsen

**Affiliations:** Experimental Psychology Unit, Faculty of Humanities and Social Sciences, Helmut Schmidt University/University of the Federal Armed Forces HamburgHamburg, Germany

**Keywords:** neuroaesthetics, aesthetic appreciation, experimental aesthetics, theory of mind (ToM), arts, event-related potential (ERP), mental chronometry

## Abstract

We used event-related brain potentials to explore the impact of mental perspective taking on processes of aesthetic appreciation of visual art. Participants (non-experts) were first presented with information about the life and attitudes of a fictitious artist. Subsequently, they were cued trial-wise to make an aesthetic judgment regarding an image depicting a piece of abstract art either from their own perspective or from the imagined perspective of the fictitious artist [i.e., theory of mind (ToM) condition]. Positive self-referential judgments were made more quickly and negative self-referential judgments were made more slowly than the corresponding judgments from the imagined perspective. Event-related potential analyses revealed significant differences between the two tasks both within the preparation period (i.e., during the cue-stimulus interval) and within the stimulus presentation period. For the ToM condition we observed a relative centro-parietal negativity during the preparation period (700–330 ms preceding picture onset) and a relative centro-parietal positivity during the stimulus presentation period (700–1100 ms after stimulus onset). These findings suggest that different subprocesses are involved in aesthetic appreciation and judgment of visual abstract art from one’s own vs. from another person’s perspective.

## Introduction

The role of cognitive processes in the aesthetic appreciation of visual artistic objects has been discussed within both philosophical and psychological frameworks. Philosophical accounts range from positing a disinterested aesthetic attitude and excluding cognitive processes from the aesthetic experience (Stolnitz, 1960) to the belief that thought and reason (especially understanding of an artwork’s symbolism) are crucial for an aesthetic experience to occur (Goodman, 1985). Recent accounts from biopsychological research indicate that aesthetic appreciation comprises both cognitive and affective components.

The aesthetic experience of a piece of art—for example, an artwork in a museum—is believed to evolve from complex interactions between specifics of the artwork (e.g., style, content, structural organization such as symmetry), the observer (knowledge about art and experience with art-related topics, i.e., expert and lay knowledge, personality traits, cognitive abilities), and the context in which this experience takes place (e.g., a natural environment, a museum, or an experimental lab, which result in different schema activations). To date, several stimulus-, person-, and situation-related factors influencing aesthetic experience and aesthetic behavior have been identified (for an overview see, e.g., Jacobsen, 2010) and assembled within a framework for the psychology of aesthetics (Jacobsen, 2006). Accordingly, aesthetic experience can be investigated from the following seven vantage points: mind, body, content, person, situation, diachronia, and ipsichronia. Each of these is regarded as featuring different levels of analysis, and they are not considered to be mutually exclusive. In the present study, we will emphasize the mind and body perspectives, as these lie at the heart of neuroaesthetics. These two vantage points constitute the scientific paradigm adopted by contemporary cognitive neuroscience and biological psychology, in which theoretical models from cognitive psychology are applied to functional neuroanatomy endeavors. Studies on aesthetic processing correlate objective measures of neural activity with reports of individual subjective experience, and are thus deeply rooted in the classical, pragmatically dualistic approach of psychophysics (Fechner, 1860).

Aesthetic experience of a visual artistic object appears to comprise two phases (Locher et al., 2007). Within the first few glances at an artwork, the beholder generates a global impression of the artwork, more specifically, a sense of its pictorial content, structural organization, and semantic meaning, and an initial affective response to it. If the initially perceived pictorial content holds interest for the beholder, the second phase will ensue, and further artwork-related perceptual and semantic information will be conceived in order to satisfy curiosity and to develop aesthetic appreciation. Studies investigating the aesthetic experience of visual artistic objects differ with respect to the role of fixation patterns. A multitude of those studies use eye movement patterns as a measure that reflects cognitive processing and indexes task performance. In contrast, in the present event-related potential (ERP) study, single fixations are used and eye movements are treated as artifacts in the electroencephalogram (EEG) recording. Leder et al. (2004) have presented a model of information-processing stages that allows for an integration of the sensory, perceptual, and cognitive processes thought to occur across the two phases involved in aesthetically appreciating and judging abstract art. These authors discuss how the cognitive processing of art generates affective aesthetic processing, which is often positive and self-rewarding, and they consider the underlying cognitive and affective processes to be somehow art-specific and reciprocally linked. According to their model, there are five cognitive processing stages: perception, explicit classification, implicit classification, cognitive mastering, and evaluation. Additionally, Leder et al. (2004) propose important variables that affect the processes at each of the five stages. The output of the modulated aesthetic processing comprises aesthetic emotions and aesthetic judgments.

In the present study, we will focus on domain-specific processing of visual modern art as follows. The stages of Leder et al.’s (2004) model that are of interest for this study will be further characterized below. The model’s cognitive mastering stage (which includes “art-specific interpretation” and “self-related interpretation”) and its evaluation stage (specifically, the “cognitive state” involved) along with the subsequent aesthetic judgment (one of two results of the evaluation of the cognitive mastering stage) are of importance for our objective, and in this model cognitive mastering and evaluation form a feedback loop. Leder et al. (2004) assume that in order to easily acquire an understanding of an artwork, self-related cognitive information (e.g., associations of the artwork’s content with the beholder’s own situation and emotional state) must be formed. In Martindale’s (1984) terms, processes eliciting aesthetic pleasure and understanding are reflected by the number and diversity of stimulus-induced semantic associations and their episodic memory associations, with a positive correlation between the number of activated cognitive units and the resulting aesthetic preference.

Leder et al. (2004) linked their cognitive mastering stage to Parsons’s (1987) proposed five-stage model of aesthetic development. For the present research endeavor, the most relevant part of Parsons’s (1987) model is stage 3, *expressiveness*. This rests on an “awareness of the interiority of the experience of others [i.e., the artist], and […] [an] ability to grasp their particular thoughts and feelings,” and it involves “a corresponding awareness of one’s own experience as something inward and unique” (Parsons, 1987, pp. 23–24). According to Parsons (1987), people can respond differently to the same artwork because of their differing understanding of it. The latter is often due to implicit specific expectations regarding the envisaged function of a piece of art, its potential inherent qualities, and feasible judgments of it. Unlike Leder et al.’s (2004) model, Parsons’s (1987) cognitive developmental model suggests that the five stages occur in a certain order, with each step being an advance over the previous one (allowing for a more adequate understanding of art), and it does not provide for feedback loops or a reversed order of the stages. Parsons (1987) proposed that factors such as the kinds of art encountered and the extent of encouragement to think about them determine where beholders wind up in the sequence of stages. Leder et al. (2004) argued that what makes an experience aesthetic might be its longer temporal extension that allows for several cycles of feedback and feedforward information flow between perception-, cognition-, and emotion-related processes. Thus, Leder et al.’s (2004) model raises questions regarding order and carry-over effects (Leder and Nadal, 2014).

Visual artistic objects can be classified along various dimensions. Some have visual qualities that are likely to elicit pleasurable aesthetic experiences. Visual stimuli such as paintings or natural landscapes may be among these. In contrast, other objects of visual art such as conceptual artworks (e.g., *objets trouvés*, *ready-mades*, much represented at modern art exhibitions like *Documenta*) might not be prone to generate a state of aesthetic pleasure by virtue of their visual qualities alone. Rather, such objects might engage aesthetic appreciation in terms of an affective experience only as a consequence of further elaboration on thoughts about the artist’s mental state, that is, his or her intentions and mental representations during artistic creation. Therefore, we propose that this attribution of mental states to others—that is, theory of mind (ToM) processing—while engaging in art appreciation is an important, if not essential, subprocess in some episodes. Such processing can enhance the depth and comprehensiveness of the aesthetic experience in cases of the former type of art object and can add aesthetic appreciation in the first place to perceptual processing in cases of the latter type.

Previous research on neural correlates of aesthetic processing points out that several general mechanisms are employed, namely, processes of perception, attention, memory, decision-making, reward, and emotion (Chatterjee, 2004; Leder et al., 2004). Given current knowledge about the neural correlates of those processes, it follows that dynamic interactions of multiple brain regions in different time frames must account for the emergence of an aesthetic experience (Jacobsen, 2006; Nadal and Pearce, 2011). However, to date the neural sources and time frames for the brain regions that specifically contribute to this particular network are less charted. In particular, little research has been aimed at investigating the multiple processing stages and mental chronometry of the processes underlying aesthetic appreciation. Such an investigation is best achieved by using ERPs derived from an EEG on the basis of its high temporal resolution.

In their work in the field of experimental aesthetics, Jacobsen and Höfel (2002) and Höfel and Jacobsen (2003) have examined the cognitive mechanisms that lead to aesthetic judgment of stimuli in the fields of visual art and music. Empirical findings suggest that the underlying conceptual structure of aesthetic experience differs across the two content domains (e.g., Jacobsen et al., 2004; Istók et al., 2009; Augustin et al., 2011). Here we present only research findings for the visual content domain, focussing on electrophysiological study results. Höfel and Jacobsen (2001) and Jacobsen and Höfel (2003) had participants judge abstract black and white graphical patterns within both an evaluative aesthetic judgment task and a descriptive symmetry judgment task (for another instantiation of an evaluative and pragmatic task see, Cupchik et al., 2009). The results of their studies showed a double dissociation in the temporal course and the neural sources for both tasks, indicating the existence of two separate processing stages (Höfel and Jacobsen, 2001, 2007a,b; Jacobsen and Höfel, 2003); On the one hand, this was construed from the ERP results for the aesthetic judgment task, showing first a fronto-central negativity at 300–400 ms and later a positive potential (LPP) peaking at 600 ms after stimulus onset (Höfel and Jacobsen, 2001; Jacobsen and Höfel, 2003). The first deflection was interpreted to reflect processes of impression formation, as the LPP has been reported to represent evaluative categorization (Cacioppo et al., 1993). On the other hand, symmetry judgments resulted in a late longer-lasting posterior ERP deflection, which was taken to reflect sustained symmetry analysis. The main findings of the 2003 study were replicated in a later study that investigated aesthetic appreciation of faces (Roye et al., 2008).

In another follow-up study, using the same abstract graphical stimulus material, the authors aimed at examining whether the pattern of results arose due to judgment categorization or judgment report. Participants in this study were asked to misreport their true judgments (false judgment task). As only the deflection of the LPP was affected by the false judgment task (in contrast to the qualitatively unaffected earlier fronto-central effect, which only showed a 100-ms-later appearance than in the 2003 study), it was concluded that both ERP deflections represent separate processing stages of the aesthetic judgment of graphic stimuli and that the observed LPP mainly reflects evaluative judgment categorization rather than judgment report (Höfel and Jacobsen, 2007a). In a further study, Höfel and Jacobsen (2007b) were able to demonstrate that the early fronto-central deflection was contingent on the intention to make an aesthetic judgment and that mere aesthetic contemplation did not result in this deflection.

Relating the findings of these studies to Leder et al.’s (2004) model, it can be assumed that the assessed ERPs reflecting two separate processing stages involved in aesthetic judgment of the beauty of abstract graphic stimuli correspond to later top-down processing stages within this model. More precisely, it could be argued that, dependent on knowledge and personal taste, the first impression formation (e.g., a beautiful vs. not beautiful graphic pattern) could correspond to the cognitive mastering stage reflected by specific fronto-central ERP deflections around 300–400 ms after stimulus onset. As to the proposed evaluative categorization process later reflected by the LPP at around 440–840 ms after stimulus onset, it remains unclear whether this corresponds to the model’s evaluation stage itself or to the subsequent aesthetic judgment. It is not yet specified whether the aesthetic judgment aspect of the model relates to a non-overt or an overt judgment within the beholder. This needs to be resolved in order to integrate those ERP effects reflecting judgment categorization and not judgment report into the model. Another recent study that used magnetoencephalography (MEG) strengthens the notion of two main processing stages in aesthetic appreciation. Therein, Cela-Conde et al. (2013) found different neural patterns following presentation of art stimuli within an earlier time window (250–750 ms, associated with an initial perceptual and semantic analysis) and within a delayed time window (1000–1500 ms, linked to a more detailed cognitive appraisal of the artwork).

To the best of our knowledge, research relating aesthetics and ToM has not yet been conducted. As an important prerequisite for understanding a wide range of social situations and interactions, quite a few studies have already focused on ToM. Reasoning about others’ mental states requires various functional processes and a reliable set of brain regions constituting a ToM network (Frith and Frith, 2003; Carrington and Bailey, 2009; McCleery et al., 2011). Empirical findings from electrophysiological and imaging studies suggest three important brain regions: the temporo-parietal junction (TPJ), the posterior superior temporal sulcus (STS), and, most consistently, the medial prefrontal cortex (mPFC; Gallagher et al., 2000; Vogeley et al., 2001; Frith and Frith, 2003; Saxe et al., 2004). In this context, the latter has been linked with the processes of calculating and representing both self- and other-perspectives.

Electrophysiological studies on ToM processing still constitute a smaller part of this research field. Additionally, these studies often focus on testing ToM development in children rather than mental chronometry in adults. ERP studies on healthy adults have mostly adopted true- vs. false-belief reasoning tasks in contrast to one or two control tasks that don’t involve ToM reasoning. For the belief/ToM tasks in question, these studies show late ERP deflections (with either positivity or negativity) time-locked to stimulus onset over anterior areas (Liu et al., 2009a: late slow wave [LSW] peaking at 800 ms; Liu et al., 2004: 700–900 ms; Liu et al., 2009b: 775–850 ms; Sabbagh and Taylor, 2000: 300–400 ms, 600–840 ms; Wang et al., 2008: 400–800 ms; Wang et al., 2010: 300–1500 ms) and posterior areas (Liu et al., 2009a: right posterior, LSW 600–800 ms; Sabbagh and Taylor, 2000: parietal, 300–400 ms, 600–840 ms). McCleery et al. (2011) investigated visual perspective taking by asking participants to judge the correctness of the number of disks seen in a picture from either the self- or the other/avatar-perspective. For the early frontal cortex component (FL190), they found longer latencies for self- than for other-judgments over the right hemisphere and the reverse pattern over the left hemisphere, as well as larger amplitudes for self-judgments on inconsistent vs. consistent trials over the right hemisphere only. Additionally, they observed a TP450 component over posterior electrodes with longer latencies for other- vs. self-judgments, larger amplitudes for consistent vs. inconsistent trials that were larger over the right hemisphere than over the left hemisphere, and a right lateralization for self-inconsistent trials only (all other conditions showing a more bilateral distribution). Similar to the aforementioned studies on true- vs. false-belief reasoning tasks, McCleery et al. (2011) also observed an LSW around 600–800 ms over frontal sites, with mean amplitudes differing for consistent vs. inconsistent trials over the right hemisphere. The authors suggested that the latter possibly reflects executive functions for the selection of perspectives by means of inhibitory control.

The ability to discriminate the self’s emotions and mental representations from those of others is a key aspect of social cognition and crucial for successful social interaction and individual well-being (e.g., Decety and Sommerville, 2003). Electrophysiological studies investigating the distinction between self- and other-perspectives have been conducted only within the last decade. A set of ERP studies on self-referential processing of stimuli such as faces, names, objects, or different word classes have stressed the notion that the P300 component is frequently associated with self–other discrimination (for an overview, see Knyazev, 2013). The P300 component represents a response to stimuli that are unexpected, salient, or motivationally relevant (salience detection; Polich and Kok, 1995). Herbert et al. (2011) investigated the temporal processing stages of self–other discrimination when silently reading unpleasant, pleasant, and neutral verbal stimuli. They found that stimulus emotionality enhanced the ERP amplitudes between 200–300 ms and 300–400 ms at parieto-occipital electrodes and that self-related unpleasant stimuli (compared to other- or unrelated unpleasant stimuli) involved a larger frontal negativity from 350 ms onward. In addition, a larger positivity over parietal electrodes has been observed for pleasant vs. unpleasant or neutral words from 450 ms onward. The authors reasoned that for verbal emotional stimuli, self–other discrimination occurs at higher-order, cortical processing stages.

Other studies have suggested an even earlier modulation of ERP components by self-relevance. Using two-sentence social vignettes with pleasant, unpleasant, and neutral emotional qualities that were presented from the third- or second-person perspective, Fields and Kuperberg (2012) observed an early modulation of P1, N1, and P2 by a self-relevant context, suggesting top-down attentional effects on early visual processing. Moreover, the results revealed an LPP (500–800 ms) that was larger for unpleasant than for pleasant words and larger for pleasant than for neutral words. There was also an effect of self-relevance on neutral words (larger LPP for self- vs. non-self-relevant words), but not on emotional words.

Luo et al. (2013) examined the brain responses of counselors and control participants to unpleasant and neutral pictures. Participants were asked to watch the pictures from either the self- or the other-perspective and to identify the content of the pictures (with or without human) by pressing a button (attention assurance task). The behavioral results revealed longer reaction times (RTs) for the other- vs. the self-perspective and longer RTs for unpleasant vs. neutral pictures. RTs did not significantly interact with stimulus and perspective. Irrespective of the stimulus type, the results showed significant differences between the two perspectives. Specifically, they revealed an N2 component over fronto-central regions with a larger amplitude for the other- vs. self-perspective (both groups) and a late positive potential (LPP) over centro-parietal regions with a larger amplitude for the self- vs. other-perspective (the difference occurred only in the counselor group). Compared to neutral pictures, unpleasant pictures elicited smaller N2 and larger LPP activations in both groups. According to Luo et al. (2013), this pattern suggests that the counselors’ self–other distinction began at an early automatic processing stage and that they were able to maintain it through the cognitive processing stage.

In some aesthetic episodes, reasoning about the artist’s mental states will be an essential part of the beholder’s interaction with an artwork. To date it remains unclear how exactly this kind of processing could be integrated in the theoretical models that are outlined above. Under which circumstances and in which way it will shape the aesthetic responses and aesthetic emotions is just as little known. In addition it would be interesting to fathom if ToM processing is also an important subprocess in other content domains than visual art (e.g., music, literature, design). In view of the empirical findings of both experimental aesthetics and ToM research (and the lack of studies combining both research lines) it is also not admissible yet to derive a specific time frame for ToM to occur in the aesthetic episode, though ERPs reflecting higher-order cognitive processing do indicate later potentials.

In the present study, we investigated the role of mental perspective taking in processes of aesthetic appreciation of visual art in a trial-by-trial cuing protocol. Using ERPs derived from the EEG as a dependent measure, we employed a scenario approach. In one condition, ToM processing was induced through an initial familiarization of the participants with a fictitious artist via narrative text. In this condition, participants made aesthetic judgments of the images from the perspective of the imagined artist. In the other condition, participants made aesthetic judgments of the images based on their own self-referential thinking. Abstract art images were chosen as stimulus material. None contained human figures or any other determinable objects, to avoid potentially confounding face or object recognition effects in the cortical activity. We predicted that preparation for target processing would differ for the ToM and non-ToM conditions, and also that the ERPs might reflect different subprocesses in the ToM and non-ToM conditions as well as their mental chronometry.

## Materials And Method

### Participants

Twelve volunteers participated in the study (1 female, 11 male; mean age 25.33 years; SD ± 2.57). All participants were students of non-art subjects and received course credit. They all reported normal or corrected-to-normal visual acuity, and no known psychiatric, neurological, or other medical problems. The right-handedness of all participants was assessed using an inventory adopted from Oldfield (1971). Prior to the experiment, they received written information about the study procedure and gave informed written consent. The study was conducted in accordance with the ethical guidelines of the German Psychological Society (Deutsche Gesellschaft für Psychologie) and the World Medical et al. (2008; Declaration of Helsinki). Formal ethics approvals for the described kind of research are not required by the guidelines of the German Psychological Association or the World Medical Association. Three additional participants were excluded from further analysis due to technical problems (online processing channel distortions) or high rejection statistics.

### Apparatus and Stimuli

During the training phases and experimental sessions, the stimuli were generated by an IBM-compatible computer running MATLAB R2010a and Psychtoolbox 3 software and were presented on a 24-inch LCD Monitor. All sessions were conducted in an electrically shielded and sound-attenuated experimental chamber (Industrial Acoustics Company GmbH, Niederkrüchten, Germany). Recording of electrophysiological data was done on a separate computer running BrainVision Recorder 1.2 (Brain Products EEG/fMRI hardware). Oﬄine signal processing was realized on a Linux-based computer running EEProbe 3.3.122 software (ANT Neuro). In addition to electrophysiological and stimulus–response data, reaction times, and missing response data were logged in order to measure performance but were not included in the later ERP analysis.

Participants were seated at a distance of approximately 1.5 m at eye height in front of the monitor, resulting in a diagonal visual angle of 3°. The abstract images were presented on a dark gray background in the center of the screen. To make a response judgment, participants were required to press one of two response keys (yes/no). They were given a rectangular keyboard (10 cm × 18 cm; response time resolution <1 ms time accuracy) with 1.0 cm × 1.0 cm response keys set 8.0 cm apart (positioned parallel to the long axis of the keyboard, with left–right key assignment counterbalanced across participants). Responses were made with the index fingers of the uncrossed left and right hands, indicating either liking (“gefällt”, via yes button) or not liking (“gefällt nicht”, via no button) in both the self- and the artist-condition.

**FIGURE 1:**
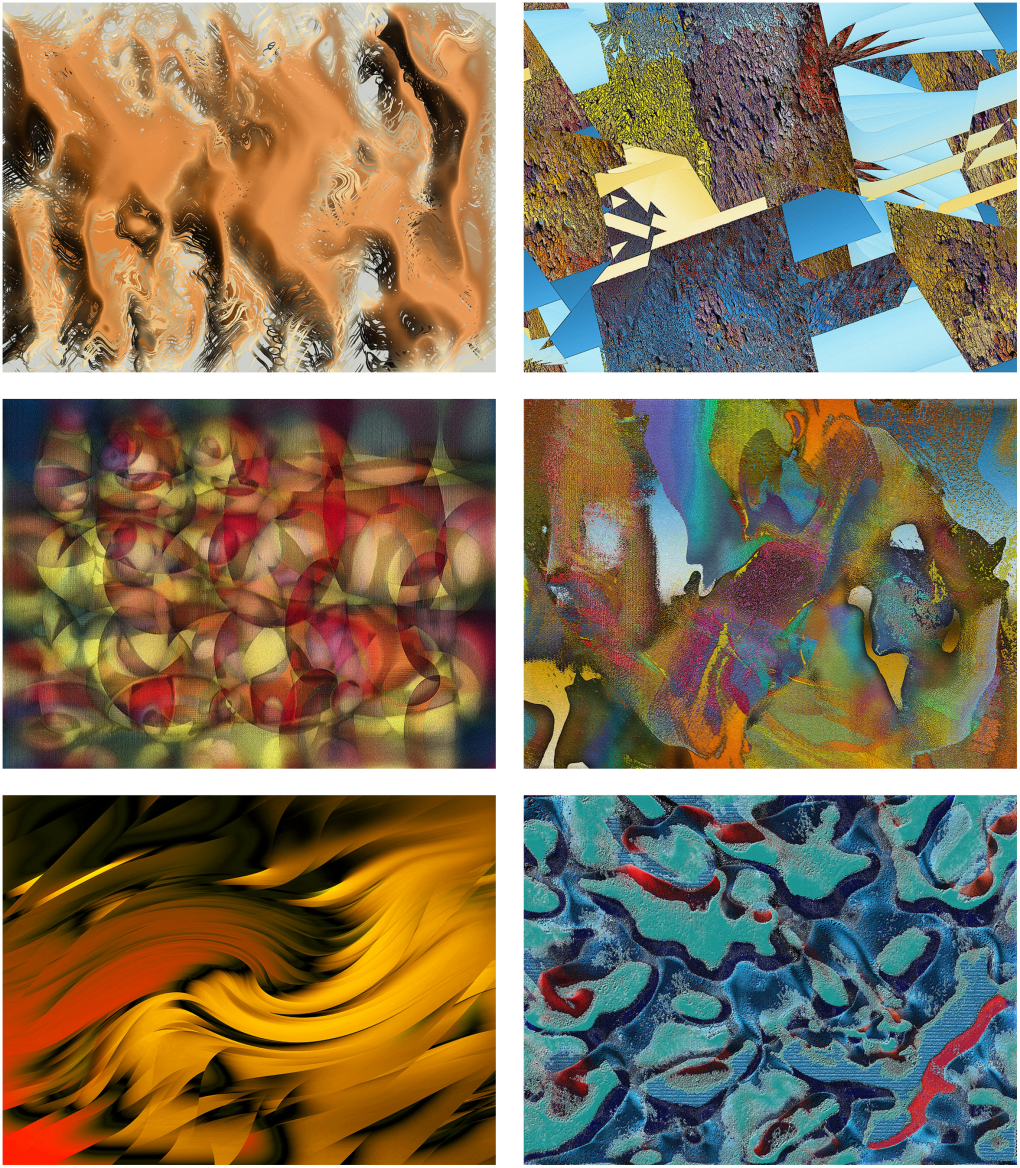
**Examples of stimuli used in the study.** Images by © Reinhard Scheiblich.

The stimulus set consisted of 290 abstract digital images (40 for practice trials, 250 for experimental trials). Of the images, 110 were provided by a local photographer and artist and 180 were taken from open source web sites. The stimuli were adjusted to the same dimensions. All pictures were colored and entirely abstract in nature. **Figure 1** illustrates some sample stimuli.

### Experimental Design and Procedure

**FIGURE 2:**
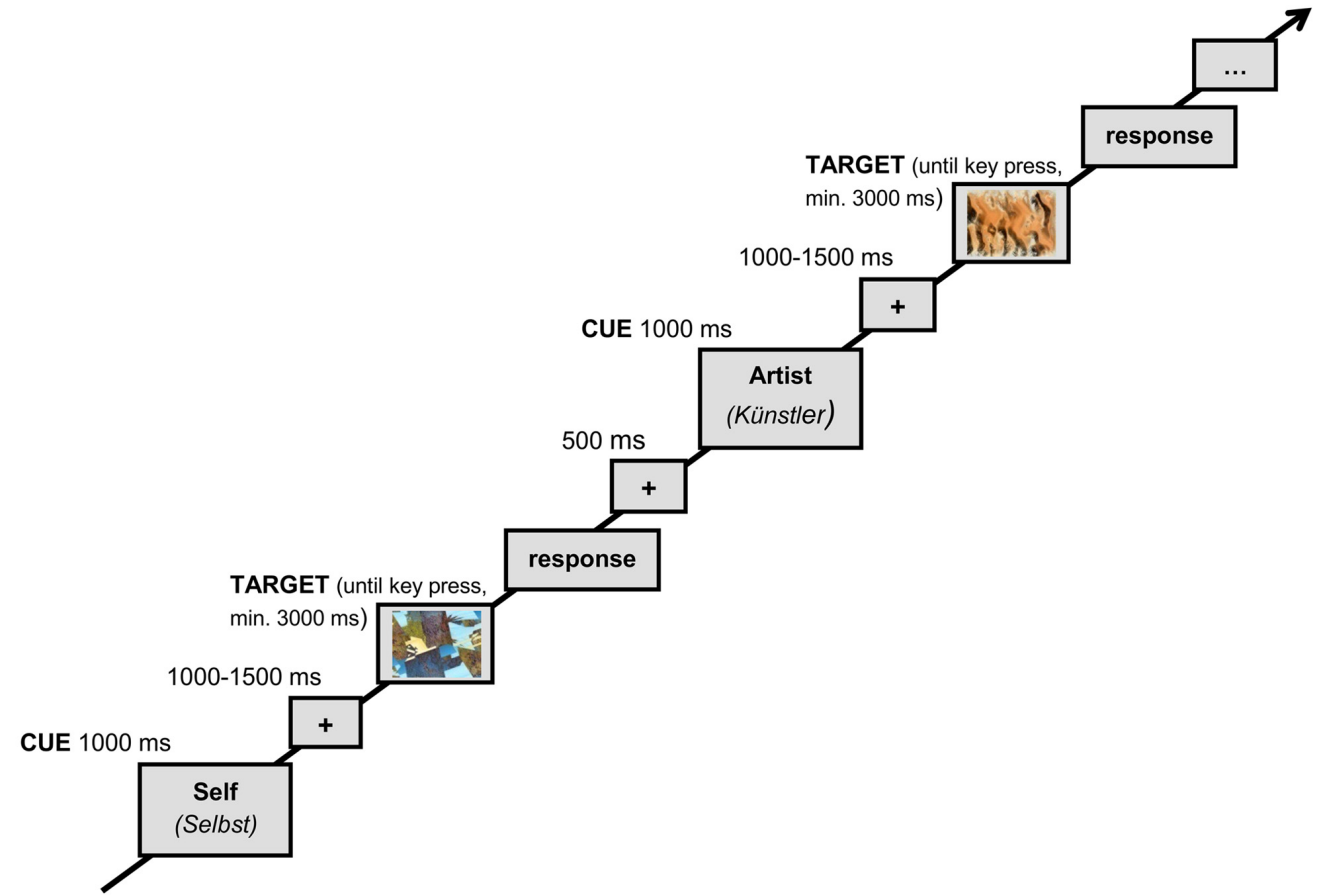
**Schematic demonstration of the experimental procedure**.

At the beginning of the experiment the participants were asked to carefully read the instructions for the following procedure, presented on the screen in front of them and initially asking them to imagine a certain artist X (“Künstler X”). Further instructions included a biographical sketch of this fictional artist which brought into focus important life events and distinct personality traits (see Supplementary Material for biographical sketch). The descriptive text was slightly inspired by anecdotal psychoanalytic narrations about various artists in Hagman (2010) and by a study of creative style and personality by Gelade (2002). The German version of the biographical sketch comprises 513 words and the whole text was presented on two instruction slides. Afterward the participants were asked whether they were able to place themselves in the position of the artist. They were further instructed about the two conditions of the experiment, self (“Selbst”; non-ToM) and artist (“Künstler”; ToM), in which they had to decide whether a presented abstract image was liked/not liked by themselves or by the artist, respectively. Participants were not told that the presented artworks were the work of the introduced artist X. Thus, within the ToM condition they were supposed to aesthetically judge artworks of other unspecified artists from the perspective of artist X. They also learned about the corresponding cues (self [Selbst]/artist [Künstler]) that indicated the following trial’s condition, and they were given an opportunity to practice the experimental procedure with a 40-trial practice block containing 10 trials for each condition (self, artist) followed by 20 trials with mixed conditions. Finally, five blocks of 50 trials each, with the experimental condition (and therefore the participant’s task) randomly chosen for each trial, were administered. Between the experimental blocks, participants could take self-paced breaks. One experimental trial began with one of the two task cues presented for 1000 ms, indicating whether participants should judge the presented abstract art stimulus as being liked/not liked by themselves (cue: self) or as being liked/not liked by artist X (cue: artist). The latter required the participants to recall the artist description in order to place themselves in the artist’s position. The task cue was followed by an interstimulus interval (ISI) of variable duration, ranging from 1000 to 1500 ms. The subsequent presentation of the abstract art stimulus lasted at least 3000 ms, remaining on the screen until a response was given, and was followed by a 500 ms interval before the next cue appeared (see **Figure 2**). During the intervals (between either cue/stimulus or stimulus/cue), a rectangular frame filled with light gray and sized according to the greatest width and height among all used stimuli was presented, with a centered black fixation cross.

After the EEG recording was completed, participants were asked to fill out a post-experiment questionnaire. This assessed the impact of the manipulation via the artist description, the difficulty of the task and the experimental design, compliance with the given instructions, potential problems that evolved during the experiment, and the general pleasantness of the experiment. The entire session lasted about 90 min, including approximately 40 min for electrode preparation.

### Electrophysiological Recordings

The EEG (Ag/AgCl electrodes) was continuously recorded from 25 standard scalp locations according to the 10–20 system (three midline electrodes: Fz/Cz/Pz, and 11 electrodes over each hemisphere: FP1/FP2, F3/F4, F7/F8, FC1/FC2, FC5/FC6, C3/C4, CP1/CP2, CP5/CP6, P3/P4, P7/P8, O1/O2) and from the left and right mastoids. The ground electrode was placed at the FCz location, and the nose tip was used as online-reference electrode. Electroocular activity was recorded with two bipolar channels. For the vertical electrooculogram (VEOG), supra- and infraorbital electrodes of the right eye were used, and for the horizontal electrooculogram (HEOG), two electrodes placed lateral to the outer canthi of both eyes were used. Impedances were kept below 5 kΩ. All channels were amplified (with low cutoff: 0.1 Hz, high cutoff: 1000 Hz) and recorded at a 500 Hz sampling rate.

### Data Analysis

The continuous EEG records were band-pass filtered with a finite impulse response filter with the following specifications: 8719 points, critical high-pass frequency of 0.1 Hz, and critical low-pass frequency of 10 Hz (CUE interval, referring to EEG data recorded between cue onset and picture onset) and 20 Hz (PIC interval, referring to EEG data recorded between picture onset and response button press). Artifact rejection was realized with a standard deviation criterion in a sliding window of 200 ms (all channels 30 μV), resulting in an exclusion of contaminated epochs from further analysis.

Different time windows were chosen for analyzing cue and picture processing following artifact rejection. For the analysis of cue processing, epochs of 1000 ms time-locked to (and preceding) picture onset were extracted and averaged separately for each of the two conditions (ToM/non-ToM). Application of high-pass filtering prior to averaging attenuated DC offset and low-frequency components, which was used as alternative for baseline correction for the CUE interval (see e.g., Widmann et al., 2015). For the analysis of picture processing (PIC Interval), epochs of 2200 ms time-locked to (and following) picture onset, including a 200 ms pre-target stimulus/picture baseline, were extracted and averaged separately for each of the two conditions (ToM/non-ToM) and for each of the two key presses (*like*/*don’t like*). Grand averages were computed from the individual subject averages.

The ERP quantification routine comprised the following steps, with all analysis based on the individual mean amplitude in the given time window at the specified electrode locations. Visual inspection led to the identification of substantial effects, and for the corresponding time windows a repeated-measures analysis of variance (rmANOVA) was conducted. For the CUE interval, a statistical window of 700 ms to 330 ms preceding picture onset was selected, and for the PIC interval, a statistical window of 700 ms to 1100 ms after picture onset was selected. For the CUE interval, mean amplitude values were subjected to an ANOVA with repeated measures on the factors Condition (non-Tom/Tom), Anterior–posterior distribution (F-line, C-line, P-line), and Laterality (left [F3, C3, P3], midline, right [F4, C4, P4]). Additionally, a fourth factor, Answer (*like*/*don’t like*), was included in the repeated-measures ANOVA conducted for the PIC interval. Nine electrodes were used for data analysis in a 3 × 3 grid. Further analyses comprised *post hoc t*-tests to localize the effects, with adjustment for multiple pairwise comparisons using Bonferroni correction. The level of the type 1 error was set to *p* < 0.05. Where the assumption of sphericity is violated, Greenhouse-Geiser (G-G) corrected degrees of freedom and G-G epsilon (𝜀) values are reported. Only significant results involving the factor Condition are reported and discussed within the text of this article.

## Results

### Behavioral Analysis

Mean response times for the subject answer *like* were 1441 ms (*SD* = 267.12) in the ToM condition and 1683 ms (*SD* = 691.65) in the non-ToM condition, and for answer *don’t like* they were 1320 ms (*SD* = 232.62) in the ToM condition and 1180 ms (*SD* = 188.56) in the non-ToM condition. The judgment latencies were analyzed through an ANOVA with repeated measures on the two factors Answer (*like*/*don’t like*) and Condition (non-Tom/Tom), which showed a significant main effect for the factor answer (*F*_1,11_ = 11.56, *MSE* = 1.17, *p* = 0.006). *Post hoc* tests indicated a shorter mean response time for negative answers (*M* = 1250 ms) than for positive answers (*M* = 1562 ms). Overall response frequencies varied between the conditions, with *N*_like_ = 768 and *N*_*don’t like*_ = 945 for ToM and *N*_like_ = 470 and *N*_*don’t like*_ = 1297 for non-ToM. Statistical analysis revealed no significant differences between crosstab cells.

On the post-experimental questionnaire, all participants confirmed that they were able to perform the required tasks. That is, on the one hand they were able to concentrate on their own aesthetic appreciation in order to make a like/not like decision and a corresponding response for themselves while viewing the art picture (non-ToM condition), and on the other hand they were also able to place themselves in artist X’s position in order to make a like/not like decision and a corresponding response while viewing the art picture (ToM condition).

### Electrophysiological Data

#### Cue Interval

**FIGURE 3:**
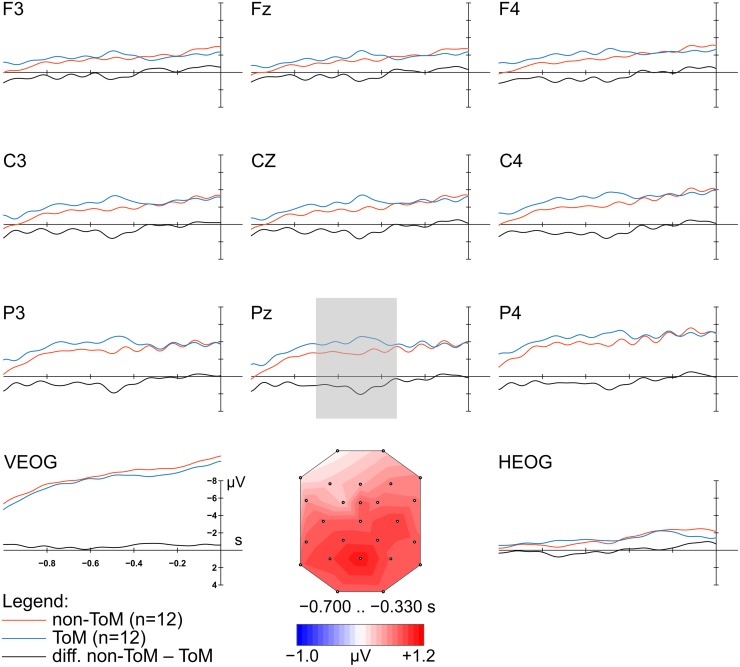
**Grand-average ERP waveforms, referenced to the nose, elicited at nine scalp electrodes and two EOG channels during preparation period time-locked to picture onset.** Voltage map representing the difference between the non-ToM and ToM condition during the time interval 0.700 to 0.330 s before picture onset. Illustrated are waveforms for the two conditions (non-ToM, ToM) and the difference wave (non-ToM-ToM). The time interval used to plot the voltage map is indicated by a gray shade (shown at electrode with highest mean amplitude difference).

Before ERP computation, on average 25.48% (ToM)/26.20% (non-ToM) of the trials per participant were rejected. On average 91 (*SD* = 17.23) and 94 (*SD* = 22.40) epochs per participant, for ToM and non-ToM conditions, respectively, were used for further analysis. For the time window between 700 and 330 ms preceding picture onset, it was tested whether the ERP responses within the two conditions, ToM and non-ToM, differed in amplitude. The repeated-measures ANOVA comprising the three factors Condition, Anterior–posterior distribution, and laterality revealed a significant main effect of the factor Condition (*F*_1,11_ = 5.18, *p* = 0.044, $\eta_{p}^{2}$ = 0.32). We observed a higher negative mean amplitude within the ToM condition (*M* = -3.04 μV) compared to the non-ToM condition (*M* = -2.24 μV). This effect was shown to be distributed differently over anterior/posterior scalp locations. The interaction effect between the factors Condition and Anterior–posterior distribution was significant (*F*_1,14_ = 7.28, *p* = 0.013, 𝜀 = 0.63, $\eta_{p}^{2}$ = 0.40). Following up on this interaction, we observed higher negative mean amplitudes within the ToM condition (*M* = -2.83 μV) compared to the non-ToM condition (*M* = -1.94 μV) for C-line and higher negative mean amplitudes within the ToM condition (*M* = -4.26 μV) compared to the non-ToM condition (*M* = -3.25 μV) for P-line (**Figure 3**). In addition, the threefold interaction of the factors Condition, Anterior–posterior distribution, and Laterality was significant (*F*_4,44_ = 2.89, *p* = 0.033, $\eta_{p}^{2}$ = 0.21).

Furthermore, the general ANOVA resulted in condition-independent main effects relating to the distribution of voltages measured across the scalp. Accordingly, there were significant main effects for Anterior–posterior distribution (*F*_1,13_ = 26.92, *p* < 0.001, 𝜀 = 0.57, $\eta_{p}^{2}$ = 0.71) and Laterality (*F*_2,22_ = 13.09, *p* < 0.001, $\eta_{p}^{2}$ = 0.54).

#### Picture Interval

**FIGURE 4:**
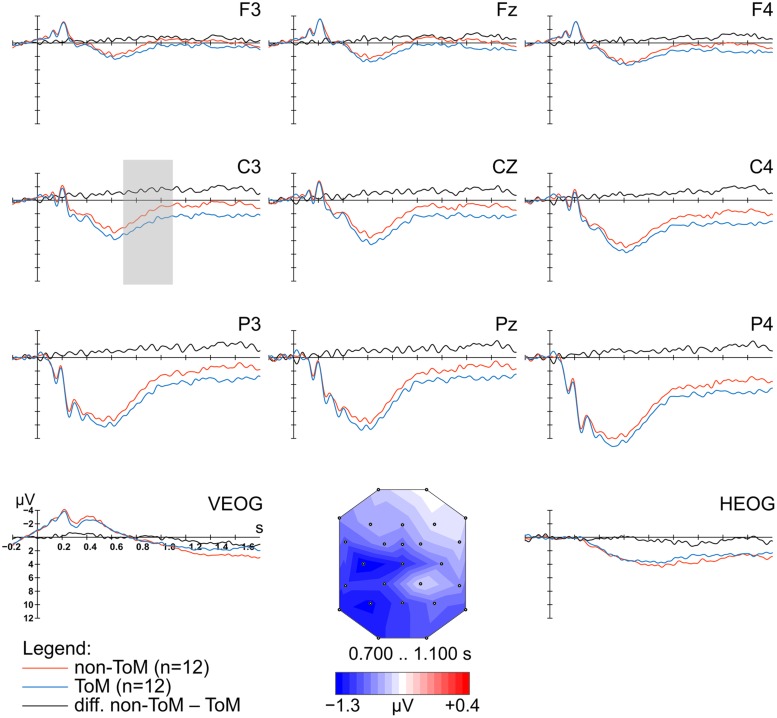
**Grand-average ERP waveforms, referenced to the nose, elicited at nine scalp electrodes and two EOG channels in response to the onset of an abstract art image, time-locked to picture onset.** Illustrated are waveforms for the two conditions (non-ToM, ToM) and the difference wave (non-ToM-ToM). The time interval used to plot the voltage map is indicated by a gray shade (shown at electrode with highest mean amplitude difference). The voltage map represents the difference between the non-ToM and ToM condition during the time interval 0.700 to 1.100 s after picture onset.

Before ERP computation, on average 19.76% (ToM)/22.27% (non-ToM) trials were rejected per participant. Thus, on average 99 (*SD* = 18.10) and 99 (*SD* = 23.50) epochs per participant, for ToM and non-ToM conditions, respectively, were used for further analysis. Based on previous findings and visual inspection, the time window 700–1100 ms after picture onset was further investigated to test for a mean amplitude difference in ERP responses between the two conditions non-ToM and ToM. A repeated-measures ANOVA comprising the four factors Condition, Answer, Anterior–posterior distribution, and Laterality was conducted for the corresponding time window. The analysis revealed a significant interaction effect for the factors Condition*Anterior–posterior distribution (*F*_2,22_ = 5.50, *p* = 0.012, $\eta_{p}^{2}$ = 0.33). Subsequent *post hoc* analyses indicated that this interaction effect arose from significant lower mean amplitude in the non-ToM condition compared to the ToM condition for central electrodes (mean difference for C-line *MD*_non-ToM-ToM_ = -1.25, *p* = 0.042) and posterior electrodes (mean difference for P-line *MD*_non-ToM-ToM_ = -1.12, *p* = 0.033). More precisely, lower mean amplitudes were found for left lateralized and central recording sites than for right hemisphere recordings (mean difference *MD*_right-left_ = -1.43, *p* = 0.001; mean difference *MD*_right-central_ = -1.42, *p* < 0.001; see **Figure 4**).

## Discussion

The present ERP study investigated the role of mental perspective taking in aesthetic appreciation of visual art. Participants were introduced to the life and attitudes of a fictitious artist via a narrative description and subsequently asked to aesthetically appraise (like/not like judgments via button presses) abstract art images either from their own perspective or from the imagined perspective of this artist. We expected that preparation for the processing of art images would differ for the ToM and non-ToM tasks. The applied technique of EEG analysis yields information about neurophysiological correlates of mental processes with a high temporal resolution, allowing for identification of subprocesses. Thus, ERPs elicited within the time window preceding response release (like/not like judgment) may reflect different subprocesses in the two conditions, along with their mental chronometry. With regard to the electrophysiological results, we obtained enhanced negative ERP waves in preparation for aesthetically appraising the abstract art images in the ToM condition. To our knowledge, the present study is the first to investigate the role of mental state attributions in the aesthetic appreciation of visual art.

### Differential Pattern for Speed of Liking Ratings

This study’s behavioral results show that the latencies of like/not like judgments differed significantly for the two evaluative judgment responses. Participants responded more quickly when they did not like the abstract art stimuli than when they liked it. Reaction times were fastest and slowest when making the evaluative judgment from the participant’s own perspective. However, the analyses revealed no significant interaction between answers and conditions, thus indicating that the difference between like/not like responses described above was observed irrespective of the adopted perspective. A similar pattern showing substantially faster judgment response for *like* vs. *don’t like* was only found when participants judged the aesthetic value and the harmonic correctness of chord sequences (Müller et al., 2010). Other studies that investigated aesthetic processing within the visual content domain and that are therefore more suitable for comparison with our study results, did not find significant differences between response latencies for *like* vs. *don’t like* responses. With regard to response latencies for the ToM vs. the control condition, empirical results show a quite heterogeneous pattern with either faster (e.g., Saxe et al., 2004; Luo et al., 2013) or slower (e.g., Kana et al., 2009) response times for the corresponding ToM condition, or even no significant differences (Wang et al., 2010). This may be partially due to very different experimental designs with varying task difficulty between the conditions, which would potentially demand different emotional and cognitive processing efforts for (successful) task fulfillment.

### Condition-dependent Modulation of Prestimulus ERPs during Preparation for Evaluative Judgment

This study showed that during the processing of the cue interval, there were significant condition-dependent differences in ERP waves. Under closer scrutiny, cue-induced adoption of another’s perspective compared to one’s own perspective elicited a stronger negativity in preparation for the upcoming abstract art stimulus and response selection. This possibly implicates that once participants become aware of the perspective they should adopt, different processing modes might be activated, depending on the perspective. More specifically, this condition-dependent difference was characterized as a slow negative-going wave over central and parietal recording sites. We suggest that these ERP waves can be associated with the contingent negative variation (CNV). This well-studied component is observed during presentation of two contingent stimuli and is perceived as an electrophysiological correlate of motor and cognitive preparatory processes. Differences in CNV amplitude have been related to factors such as differences in task difficulty and effort investment (Lorist et al., 2000; Falkenstein et al., 2003). Hence, the higher ERP negativity elicited in the ToM condition might represent a greater amount of effort required to accomplish the subsequent task. We assume that this negative deflection representing preparation processing might be even more pronounced if the cue-target ISI were set as fixed. In task-switching studies (see Kiesel et al., 2010, for an overview), preparation effects, as indexed by performance facilitation due to the elongation of an advanced preparation interval, tend to be more pronounced when the preparation intervals are kept constant rather than varied within blocks of trials (e.g., Rogers and Monsell, 1995). Thus, the jittered ISI implemented within the present study may have added to a diminished CNV deflection.

### ERP Effects of the Adopted Perspective

Preceding the participants’ motor response indicating liking or disliking of the abstract art image, we discovered a slow negative-going wave that differed between the ToM and non-ToM conditions (**Figure 4**). The negative deflection was observed to be stronger when participants were deliberating their own picture evaluation rather than the imagined evaluation of another person, i.e., the fictitious artist. This effect evolved mainly from left lateralized, central, and posterior recording sites. The neural responses associated with the different judgments (*like*/*don’t like*) did not differ substantially preceding the motor response. Thus, the difference between the ERP responses for the two perspective conditions did not seem to be modulated by the kind of the corresponding evaluative judgment.

In contrast, previous ERP studies investigating the chronometry of aesthetic processing have reported slow late positive-going potentials within similar time windows for the aesthetic evaluation task (e.g., music: Brattico et al., 2010, 2013; abstract graphic patterns: Jacobsen and Höfel, 2003). However, visual inspection of their presented grand-average ERP waves indicates that the reported LPPs seemed to be followed by a sustained negativity as well. For the latter potential, neither significant differences between like and dislike answers nor between different judgment tasks—aesthetic judgment tasks versus correctness judgments (Brattico et al., 2010, 2013) or versus symmetry judgment tasks (Jacobsen and Höfel, 2003)—could be reported.

Late negative ERP effects have also rarely been reported in studies investigating the neural time course of ToM processing and, more specifically, the differences between belief reasoning and reality reasoning. Wang et al. (2008) used electrophysiological recording to investigate neural substrates of false-belief versus true-belief reasoning in a deceptive appearance task. Compared with true-belief reasoning, for which information can simply be derived from reality with no ToM reasoning required to accomplish the task, a significantly decreased amplitude of the late negative component (LNC, 400–800 ms) over centro-frontal sites was elicited by false-belief reasoning (for which ToM reasoning is required). Drawing parallels between their task design and the two tasks that were integrated in the present study, it can be suggested that in both studies, there is a decreased amplitude of the late negative component for the ToM perspective compared to the self- or reality-perspective. It must be noted that in the present study, differences were obtained at centro-parietal sites. Wang et al. (2008) concluded that in their study the brain was not able to differentiate true- and false-belief reasoning before 400 ms after stimulus onset and that the subsequent decreased amplitude may reflect a decoupling mechanism that distinguishes between mental states and reality (also see Liu et al., 2004). Given this, the ToM and non-ToM tasks contrasted in the present study and thus also the obtained results cannot be fairly compared with the study results based on contrasting true- versus false-belief reasoning. Unlike the true/reality reasoning task, the self/non-ToM task is not characterized by decoupling from mental states, which in this case are thought to be self-referential. As a consequence, our own results may be better compared with the findings in Wang et al. (2010), where a person perception condition was added, which could then be contrasted with a ToM and a scene condition, thereby enabling investigation of a more difficult task discrimination (ToM and person perception) than the discrimination often found in established true- or false-belief reasoning designs. In their study, Wang et al. (2010) observed that the dissociation of ToM and person perception emerged considerably later (1000–1400 ms). They concluded that longer latencies could be explained by differences in cognitive difficulty. This may possibly be due to a greater cognitive task demand and also the difficulty of the task discrimination.

Furthermore, integrating results from imaging studies that stress the involvement of the anterior cingulate cortex (ACC) in mentalizing processes (e.g., Gallagher et al., 2000, 2002; Vogeley et al., 2001), and the acknowledged involvement of the ACC in inhibitory processing (e.g., Weissman et al., 2003; Botvinick et al., 2004) with ERP results found by Wang et al. (2010), the latter suggested that their LNC represents the crucial role of inhibition in false-belief (ToM) reasoning, through inhibiting self-predominating thoughts. Given that, the late negativity effect in the present study can also be considered as a decreased negative slow potential when the ToM versus the non-ToM perspective is adopted, thereby representing the activation of inhibitory processes to limit the processing of self-referential mental states.

The comparability of the present study’s results to previous experiments in the corresponding research fields is restricted due to different experimental designs and different tested assumptions. Therefore, it may be argued that the specificity of conclusions drawn about the results should be considered critically. Also, given the effect size of the present results, future research might be well advised to adjust the size of the participant sample accordingly.

## Conclusion

Art reception can be accompanied by an aesthetic experience involving perceptual, cognitive, emotional, and evaluative processes. The corresponding research is only at the beginning stage of considering the possibilities that arise from the adoption of insights and methods from cognitive psychology and from cognitive and affective neuroscience. Little is known yet about how aesthetic experiences are instantiated in the brain and how information is processed over the time course of an aesthetic episode and its underlying processes. We argue that one important cognitive process substantially contributing to a subset of aesthetic experiences is ToM processing. The present study is the first to address this specific issue using a multimethod research approach involving self-reports, response times, and the ERP technique. Substantial differences in ERP mean amplitudes for the ToM and self-perspective conditions were found both in preparation for appreciating an art image and later preceding an overt aesthetic judgment. We take our results to reflect a higher cognitive demand when preparing for aesthetic appreciation from another’s perspective, and to indicate different subprocesses underlying aesthetic appreciation of visual art during the modes of mental state attribution and self-referential processing. Mentalizing processes occur in an extended time window between initial perceptual and eventual decision processes.

## Conflict of Interest Statement

The authors declare that the research was conducted in the absence of any commercial or financial relationships that could be construed as a potential conflict of interest.

## References

[B1] Augustin M. D. Defranceschi B. Fuchs H. K. Carbon C.-C. Hutzler F. (2011). The neural time course of art perception: an ERP study on the processing of style versus content in art. *Neuropsychologia* 49 2071–2081. 10.1016/j.neuropsychologia.2011.03.038 21477603

[B2] Botvinick M. M. Cohen J. D. Carter C. S. (2004). Conflict monitoring and anterior cingulate cortex: an update. *Trends Cogn. Sci.* 8 539–546. 10.1016/j.tics.2004.10.003 15556023

[B3] Brattico E. Bogert B. Jacobsen T. (2013). Toward a neural chronometry for the aesthetic experience of music. *Front. Psychol.* 4:206 10.3389/fpsyg.2013.00206 PMC3640187 23641223

[B4] Brattico E. Jacobsen T. de Baene W. Glerean E. Tervaniemi M. (2010). Cognitive vs. affective listening modes and judgments of music: an ERP study. *Biol. Psychol.* 85 393–409. 10.1016/j.biopsycho.2010.08.014 20837091

[B5] Cacioppo J. T. Crites S. L. Berntson G. G. Coles M. G. (1993). If attitudes affect how stimuli are processed, should they not affect the event-related brain potential? *Psychol. Sci.* 4 108–112. 10.1111/j.1467-9280.1993.tb00470.x

[B6] Carrington S. J. Bailey A. J. (2009). Are there theory of mind regions in the brain? A review of the neuroimaging literature. *Hum. Brain Mapp.* 30 2313–2335. 10.1002/hbm.2067 19034900 PMC6871093

[B7] Cela-Conde C. J. García-Prieto J. Ramasco J. J. Mirasso C. R. Bajo R. Munar E. (2013). Dynamics of brain networks in the aesthetic appreciation. *Proc. Natl. Acad. Sci. U.S.A.* 110 10454–10461. 10.1073/pnas.1302855110 23754437 PMC3690613

[B8] Chatterjee A. (2004). The neuropsychology of visual artistic production. *Neuropsychologia* 42 1568–1583. 10.1016/j.neuropsychologia.2004.03.011 15246293

[B9] Cupchik G. C. Vartanian O. Crawley A. Mikulis D. J. (2009). Viewing artworks: contributions of cognitive control and perceptual facilitation to aesthetic experience. *Brain Cogn.* 70 84–91. 10.1016/j.bandc.2009.01.003 19223099

[B10] Decety J. Sommerville J. A. (2003). Shared representations between self and other: a social cognitive neuroscience view. *Trends Cogn. Sci.* 7 527–533. 10.1016/j.tics.2003.10.004 14643368

[B11] Falkenstein M. Hoormann J. Hohnsbein J. Kleinsorge T. (2003). Short-term mobilization of processing resources is revealed in the event-related potential. *Psychophysiology* 40 914–923. 10.1111/1469-8986.00109 14986844

[B12] Fechner G. T. (1860). *Elemente der Psychophysik*. Leipzig: Breitkopf & Härtel.

[B13] Fields E. C. Kuperberg G. R. (2012). It’s all about you: an ERP study of emotion and self-relevance in discourse. *Neuroimage* 62 562–574. 10.1016/j.neuroimage.2012.05.003 22584232 PMC3678961

[B14] Frith U. Frith C. D. (2003). Development and neurophysiology of mentalizing. *Philos. Trans. R. Soc. Lond. B Biol. Sci.* 358 459–473. 10.1098/rstb.2002.1218 12689373 PMC1693139

[B15] Gallagher H. L. Happé F. Brunswick N. Fletcher P. C. Frith U. Frith C. D. (2000). Reading the mind in cartoons and stories: an fMRI study of ‘theory of mind’ in verbal and nonverbal tasks. *Neuropsychologia* 38 11–21. 10.1016/S0028-3932(99)00053-6 10617288

[B16] Gallagher H. L. Jack A. I. Roepstorff A. Frith C. D. (2002). Imaging the intentional stance in a competitive game. *Neuroimage* 16 814–821. 10.1006/nimg.2002.1117 12169265

[B17] Gelade G. A. (2002). Creative style, personality, and artistic endeavor. *Genet. Soc. Gen. Psychol. Monogr.* 128 213–234. 12401033

[B18] Goodman N. (1985). *Languages of Art: An Approach to a Theory of Symbols*. Indianapolis: Hackett.

[B19] Hagman G. (2010). *The Artist’s Mind: A Psychoanalytic Perspective on Creativity, Modern Art and Modern Artists*. London: Routledge.

[B20] Herbert C. Herbert B. M. Ethofer T. Pauli P. (2011). His or mine? the time course of self–other discrimination in emotion processing. *Soc. Neurosci.* 6 277–288. 10.1080/17470919.2010.523543 21104542

[B21] Höfel L. Jacobsen T. (2001). Aesthetics electrified: an analysis of descriptive symmetry and evaluative aesthetic judgment processes using event-related brain potentials. *Empir. Stud. Arts* 19 177–190. 10.2190/P7W1-5F1F-NJK9-X05B

[B22] Höfel L. Jacobsen T. (2003). Temporal stability and consistency of aesthetic judgments of beauty of formal graphic patterns. *Percept. Mot. Skills* 96 30–32. 10.2466/pms.2003.96.1.30 12705506

[B23] Höfel L. Jacobsen T. (2007a). Electrophysiological indices of processing aesthetics: spontaneous or intentional processes? *Int. J. Psychophysiol.* 65 20–31. 10.1016/j.ijpsycho.2007.02.007 17400317

[B24] Höfel L. Jacobsen T. (2007b). Electrophysiological indices of processing symmetry and aesthetics. *J. Psychophysiol.* 21 9–21. 10.1027/0269-8803.21.1.9 17400317

[B25] Istók E. Brattico E. Jacobsen T. Krohn K. Muller M. Tervaniemi M. (2009). Aesthetic responses to music: a questionnaire study. *Music Sci.* 13 183–206. 10.1177/102986490901300201

[B26] Jacobsen T. (2006). Bridging the arts and sciences: a framework for the psychology of aesthetics. *Leonardo* 39 155–162. 10.1162/leon.2006.39.2.155

[B27] Jacobsen T. (2010). Beauty and the brain: culture, history and individual differences in aesthetic appreciation. *J. Anat.* 216 184–191. 10.1111/j.1469-7580.2009.01164.x 19929909 PMC2815941

[B28] Jacobsen T. Buchta K. Köhler M. Schröger E. (2004). The primacy of beauty in judging the aesthetics of objects. *Psychol. Rep.* 94 1253–1260. 10.2466/pr0.94.3c.1253-1260 15362400

[B29] Jacobsen T. Höfel L. (2002). Aesthetic judgments of novel graphic patterns: analyses of individual judgments. *Percept. Mot. Skills* 95 755–766. 10.2466/pms.2002.95.3.755 12509172

[B30] Jacobsen T. Höfel L. (2003). Descriptive and evaluative judgment processes: behavioral and electrophysiological indices of processing symmetry and aesthetics. *Cogn. Affect. Behav. Neurosci.* 3 289–299. 10.3758/CABN.3.4.289 15040549

[B31] Kana R. K. Keller T. A. Cherkassky V. L. Minshew N. J. Just M. A. (2009). Atypical frontal-posterior synchronization of theory of mind regions in autism during mental state attribution. *Soc. Neurosci.* 4 135–152. 10.1080/17470910802198510 18633829 PMC3086301

[B32] Kiesel A. Steinhauser M. Wendt M. Falkenstein M. Jost K. Philipp A. M. (2010). Control and interference in task switching: a review. *Psychol. Bull.* 136 849–874. 10.1037/a0019842 20804238

[B33] Knyazev G. G. (2013). EEG correlates of self-referential processing. *Front. Hum. Neurosci.* 7:264 10.3389/fnhum.2013.00264 PMC3674309 23761757

[B34] Leder H. Belke B. Oeberst A. Augustin D. (2004). A model of aesthetic appreciation and aesthetic judgments. *Br. J. Psychol.* 95(Pt 4), 489–508. 10.1348/0007126042369811 15527534

[B35] Leder H. Nadal M. (2014). Ten years of a model of aesthetic appreciation and aesthetic judgments: the aesthetic episode—developments and challenges in empirical aesthetics. *Br. J. Psychol.* 105 443–464. 10.1111/bjop.12084 25280118

[B36] Liu D. Meltzoff A. N. Wellman H. M. (2009a). Neural correlates of belief- and desire-reasoning. *Child Dev.* 80 1163–1171. 10.1111/j.1467-8624.2009.01323.x 19630900 PMC3039678

[B37] Liu D. Sabbagh M. A. Gehring W. J. Wellman H. M. (2009b). Neural correlates of children’s theory of mind development. *Child Dev.* 80 318–326. 10.1111/j.1467-8624.2009.01262.x 19466994 PMC3073354

[B38] Liu D. Sabbagh M. A. Gehring W. J. Wellman H. M. (2004). Decoupling beliefs from reality in the brain: an ERP study of theory of mind. *Neuroreport* 15 991–995. 10.1097/00001756-200404290-00012 15076721

[B39] Locher P. Krupinski E. A. Mello-Thoms C. Nodine C. F. (2007). Visual interest in pictorial art during an aesthetic experience. *Spat. Vis.* 21 55–77. 10.1163/156856807782753868 18073051

[B40] Lorist M. M. Klein M. Nieuwenhuis S. de Jong R. Mulder G. Meijman T. F. (2000). Mental fatigue and task control: planning and preparation. *Psychophysiology* 37 614–625. 10.1111/1469-8986.3750614 11037038

[B41] Luo P. Qu C. Chen X. Zheng X. Jiang Y. Zheng X. (2013). A comparison of counselors and matched controls in maintaining different brain responses to the same stimuli under the self-perspective and the other-perspective. *Brain Imaging Behav.* 7 188–195. 10.1007/s11682-012-9214-z 23242969

[B42] Martindale C. (1984). The pleasures of thought: a theory of cognitive hedonics. *J. Mind Behav.* 5 49–80.

[B43] McCleery J. P. Surtees A. D. R. Graham K. A. Richards J. E. Apperly I. A. (2011). The neural and cognitive time course of theory of mind. *J. Neurosci.* 31 12849–12854. 10.1523/JNEUROSCI.1392-11.2011 21900563 PMC3176738

[B44] Müller M. Höfel L. Brattico E. Jacobsen T. (2010). Aesthetic judgments of music in experts and laypersons: an ERP study. *Int. J. Psychophysiol.* 76 40–51. 10.1016/j.ijpsycho.2010.02.002 20153786

[B45] Nadal M. Pearce M. T. (2011). The Copenhagen neuroaesthetics conference: prospects and pitfalls for an emerging field. *Brain Cogn.* 76 172–183. 10.1016/j.bandc.2011.01.009 21334125

[B46] Oldfield R. C. (1971). The assessment and analysis of handedness: the Edinburgh inventory. *Neuropsychologia* 9 97–113. 10.1016/0028-3932(71)90067-4 5146491

[B47] Parsons M. J. (1987). *How We Understand Art: A Cognitive Developmental Account of Aesthetic Experience*. Cambridge: Cambridge University Press.

[B48] Polich J. Kok A. (1995). Cognitive and biological determinants of P300: an integrative review. *Biol. Psychol.* 41 103–146. 10.1016/0301-0511(95)05130-9 8534788

[B49] Rogers R. D. Monsell S. (1995). Costs of a predictible switch between simple cognitive tasks. *J. Exp. Psychol. Gen.* 124 207–231. 10.1037/0096-3445.124.2.207

[B50] Roye A. Höfel L. Jacobsen T. (2008). Aesthetics of faces. *J. Psychophysiol.* 22 41–57. 10.1027/0269-8803.22.1.41

[B51] Sabbagh M. A. Taylor M. (2000). Neural correlates of theory-of-mind reasoning: an event-related potential study. *Psychol. Sci.* 11 46–50. 10.1111/1467-9280.00213 11228842

[B52] Saxe R. Carey S. Kanwisher N. (2004). Understanding other minds: linking developmental psychology and functional neuroimaging. *Annu. Rev. Psychol.* 55 87–124. 10.1146/annurev.psych.55.090902.142044 14744211

[B53] Stolnitz J. (1960). *Aesthetics and Philosophy of Art Criticism: A Critical Introduction*. Boston: Houghton Miﬄin Co.

[B54] Vogeley K. Bussfeld P. Newen A. Herrmann S. Happé F. Falkai P. (2001). Mind reading: neural mechanisms of theory of mind and self-perspective. *Neuroimage* 14 (Pt 1), 170–181. 10.1006/nimg.2001.0789 11525326

[B55] Wang Y. W. Lin C. D. Yuan B. Huang L. Zhang W. X. Shen D. L. (2010). Person perception precedes theory of mind: an event related potential analysis. *Neuroscience* 170 238–246. 10.1016/j.neuroscience.2010.06.055 20603189

[B56] Wang Y. W. Liu Y. Gao Y. X. Chen J. Zhang W. Lin C. D. (2008). False belief reasoning in the brain: an ERP study. *Sci. China C Life Sci.* 51 72–79. 10.1007/s11427-008-0014-z 18176794

[B57] Weissman D. H. Giesbrecht B. Song A. W. Mangun G. R. Woldorff M. G. (2003). Conflict monitoring in the human anterior cingulate cortex during selective attention to global and local object features. *Neuroimage* 19 1361–1368. 10.1016/S1053-8119(03)00167-8 12948694

[B58] Widmann A. Schröger E. Maess B. (2015). Digital filter design for electrophysiological data - a practical approach. *J. Neurosci. Methods* 250 34–46. 10.1016/j.jneumeth.2014.08.002 25128257

[B59] World Medical Association (2008). *Declaration of Helsinki: Ethical Principles for Medical Research Involving Human Subjects*. Available at: http://www.wma.net/en/30publications/10policies/b3/17c.pdf 19886379

