# Supplementary material for: On the Role of Mentalizing Processes in Aesthetic Appreciation: An ERP Study
Source: Front Hum Neurosci. 2015 Nov 13;9:600. doi: 10.3389/fnhum.2015.00600 (PMC4643139; doi:10.3389/fnhum.2015.00600)
Supplement: Supplementary file 1 [file Data_Sheet_1.DOCX]

***Supplementary Material***

**On the role of mentalizing processes in aesthetic appreciation:**

**An ERP study**

**Susan Beudt^1^*, Thomas Jacobsen^1^**

^1^ Experimental Psychology Unit, Humanities and Social Sciences, Helmut-Schmidt-University / University of the Federal Armed Forces Hamburg, Hamburg, Germany

*** Correspondence:** Susan Beudt**,** Helmut-Schmidt-University / University of the Federal Armed Forces Hamburg, Humanities and Social Sciences, Experimental Psychology Unit

Holstenhofweg 85, Hamburg, 22043, Germany

[s.beudt@hsu-hh.de](mailto:s.beudt@hsu-hh.de)

1. **Supplementary Instruction Data (professional translation)**

**Artistic Profile of X**

X is an extremely significant contemporary painter. Together with his siblings, he grew up in a highly unstable environment. His father, an unsuccessful businessman, repeatedly suffered financial crises on account of his self-indulgent and unrealistic wife. A caring but also very busy man, the father soon left his nagging and narcissistic wife but remained in regular contact with his children.

The mother, a demanding and aesthetically sensitive woman with a fondness for culture and art, invested a great deal of effort and money in the decoration of their home and thus increased their debts. Her motherly care increasingly wavered between unduly concern and neglect – the latter caused by constant financial problems, downward social mobility, and a growing preoccupation with her own prestige. Ever more frequently, X was looked after by his siblings. They were just as incapable of responding to his fears in a caring way and considered his immaturity and neediness to be a problem. The family soon came to expect little of him and treated him like a disabled person who constantly needed support and assistance.

He only found comfort when he was with his eldest brother. He admired him and the ambition he showed at art school. Even though X showed no obvious artistic talent, he hoped to follow in his brother's footsteps. After several years of training and practice, he succeeded in entering an art school, where a renowned artist became his mentor. X dreamt of success in the world of art, which his mother loved so much.

His mother's inability to respond to his needs caused him to crave for her attention and admiration for the rest of his life. As an adult he was incapable of accepting the help of others. Stress and frustration regularly overwhelmed him and led to narcissist outbreaks of rage, which he countered with excessive alcohol consumption. After drinking even small amounts of alcohol, he would repel and rebuff people. On one occasion, he attacked the paintings of other artists; on another, he publicly urinated in a museum. His outbreaks were always different.

His mentor gave him advice and support for many years but dominated his art by insisting on the use of traditional painting techniques and an objective approach. These restrictions as well as self-doubts kept him from evolving beyond the mediocrity of his artworks for a long time. A change occurred only when he consulted a therapist who recommended that he let his inner experience flow into his art and, instead of looking for the objectively "correct" line, that he search for the line which for him best corresponded to what he felt inside.

He then entered an artistic phase in which he liberated himself from the restrictions of perspective and realism. He experimented greatly and created more playful and open paintings. He often used unusual means to apply paint such as tree roots or pieces of glass and frequently worked the canvas quickly and with brute force. Today, these works of art are considered to be highly innovative. Characterised by a massive presence and a lack of clear composition, they convey the impression of force and power. His most important works arouse in the viewer an aesthetic experience of intense and sublime beauty.Please submit any data, information, figures, or tables that are not part of the main text of the article, as supplementary material.
